# Retraction: LncRNA MALAT1 acts as an oncogene in multiple myeloma through sponging miR5095p to modulate FOXP1 expression

**DOI:** 10.18632/oncotarget.28885

**Published:** 2026-05-20

**Authors:** Yueli Gu, Xichun Xiao, Shuo Yang

**Affiliations:** ^1^Department of Hematology, The First People’s Hospital of Shangqiu, Shangqiu 476100, China

**This article has been retracted:** Oncotarget has concluded its investigation of this paper, and the following issues were discovered:

- Figure 2C: The four panels in the colony formation assay are duplicates of four panels in Figure 2C of an unrelated article [1], which was published concurrently and recently retracted.
- Figure 3A: The tumors shown are identical to those appearing in Figure 3B of reference [2] and Figure 4B of reference [3], both of which are unrelated papers from 2017.
- Figure 6C: The western blots for FOXP1 in OPM-2 and U266 cells are duplicates.
- Figure 7D: The FOXP1 western blots for OPM-2 and U266 cells are reproduced in Figure 4 of a 2018 article [4] as c-Myc and Cyclin D1, respectively.

As there has been no response to our requests for comment or the original data, the editorial decision has been made to retract this paper. We have reached out to all authors regarding this notification but have not received a reply.

Original article: Oncotarget. 2017; 8:101984–101993. 101984-101993. https://doi.org/10.18632/oncotarget.21957
